# Impact of obesity and metabolic health status in the development of non-alcoholic fatty liver disease (NAFLD): A United Kingdom population-based cohort study using the health improvement network (THIN)

**DOI:** 10.1186/s12902-020-00582-9

**Published:** 2020-06-30

**Authors:** A. Vusirikala, T. Thomas, N. Bhala, A. A. Tahrani, G. N. Thomas, K. Nirantharakumar

**Affiliations:** 1 Institute of Applied Health Research, University of Birmingham, Edgbaston, Birmingham, B15 2TT UK; 2 Translational Gastroenterology Unit, University of Oxford, Oxford, UK; 3 Kennedy Institute of Rheumatology, University of Oxford, Oxford, UK; 4 Wellcome Centre for Human Genetics, University of Oxford, Oxford, UK; 5 Queen Elizabeth Hospital Birmingham, Birmingham, UK; 6 Department of Diabetes and Endocrinology, University Hospitals Birmingham, Birmingham, UK; 7 Centre for Endocrinology, Diabetes and Metabolism (CEDAM), Birmingham Health Partners, Birmingham, UK; 8 Institute of Metabolism and Systems Research, University of Birmingham, Birmingham, UK

## Abstract

**Background:**

With the obesity epidemic reaching crisis levels, there has been attention around those who may be resilient to the effects of obesity, termed metabolically healthy obesity (MHO), who initially present without associated metabolic abnormalities. Few longitudinal studies have explored the relationship between MHO and non-alcoholic fatty liver disease (NAFLD), which we address using over 4 million primary care patient records.

**Methods:**

A retrospective population-based longitudinal cohort was conducted using The Health Improvement Network (THIN) database incorporating adults with no history of NAFLD or alcohol excess at baseline. Individuals were classified according to BMI category and metabolic abnormalities (diabetes, hypertension and dyslipidaemia). Diagnosis of NAFLD during follow-up was the primary outcome measure. NAFLD was identified by Read codes.

**Results:**

During a median follow-up period of 4.7 years, 12,867 (0.3%) incident cases of NAFLD were recorded in the cohort of 4,121,049 individuals. Compared to individuals with normal weight and no metabolic abnormalities, equivalent individuals who were overweight, or obese were at significantly greater risk of incident NAFLD (Adjusted HR 3.32 (95%CI 2.98–3.49), and 6.92 (6.40–7.48, respectively). Metabolic risk factors further increased risk, including in those with normal weight and 1 (2.27, 1.97–2.61) or = < 2 (2.39, 1.99–2.87) metabolic abnormalities.

**Conclusions:**

MHO individuals are at greater risk of developing NAFLD compared to those with normal weight. This finding supports that the MHO phenotype is a temporary state, and weight must be considered a risk factor even before other risk factors develop. Being normal weight with metabolic abnormalities was also associated with risk of NAFLD.

## Background

Non-alcoholic fatty liver disease (NAFLD) represents a major global healthcare challenge in the twenty-first century. Driven by the obesity epidemic [1], the global prevalence of NAFLD is estimated at around 25.2%, and ranges from 13.5% in Africa to as high as 31.8% in the Middle East [2, 3]. However, it is well established that there is significant variation in the presence of concurrent metabolic abnormalities such as hyperglycaemia, hypertension and hyperlipidaemia in patients with obesity. This has led to the characterisation of patients with obesity and associated metabolic abnormalities into subphenotypes. Patients with obesity, as defined by body mass index (BMI), may present without the aforementioned metabolic complications. This subpopulation has been described as “metabolically healthy obese” (MHO). In contrast, a subgroup of normal BMI individuals with metabolic abnormalities have been termed “metabolically unhealthy non-obese” (MUNO) [4, 5]. The implications for the development of NAFLD across these subphenotypes remains unclear.

Data from a recent large population-based cohort study (comprising > 3 million individuals) examining cardiovascular risk across obesity phenotypes have suggested that individuals with MHO phenotype were at higher risk of coronary heart disease (CHD), cerebrovascular disease and heart failure than “metabolically healthy normal weight” individuals [6]. The study specifically highlights that individuals with obesity but without other metabolic abnormalities were at risk of cardiovascular events, and also that individuals with risk factors regardless of BMI status were equally at increased risk, highlighting that clinicians should be aware of the risk of cardiovascular disease in patients without obesity. Similar studies investigating the development of NAFLD have led to inconclusive results [7, 8].

The majority of research to date demonstrating an association between MHO and NAFLD is from one particular cohort called the Kangbuk Samsung Health Study in South Korea. First, a study by Chang et al. [7] showed a graded dose-response relationship with increasing baseline BMI in individuals with no metabolic abnormalities and incidence of NAFLD, suggesting that obesity has a major impact on the development of NAFLD irrespective of metabolic health. This study was only limited to metabolically healthy participants. However, another smaller study (*n* = 3045) including participants from the same Korean cohort compared metabolically healthy and unhealthy individuals concluding that metabolic health is a greater determinant in developing NAFLD than obesity [8]. This study didn’t use a strict definition for metabolic health and inappropriately termed individuals as metabolically healthy if they had less than two metabolic abnormalities. Studies have indicated that even having one metabolic abnormality can increase risk of cardiovascular conditions [6, 9, 10] and therefore to accurately assess the relationship between metabolic health status and NAFLD, “metabolically healthy” should be defined as absence of any metabolic abnormality of interest.

With rates of decompensated cirrhosis and hepatocellular cancer (HCC) secondary to NAFLD estimated to double by 2030 [11] and NAFLD, a leading indication for liver transplantation (LT) [12, 13], it is increasingly important to further elucidate susceptible phenotypes for NAFLD development. Our study aimed to examine the association between different body size phenotypes with and without metabolic abnormalities and incident NAFLD.

## Methods

The study is reported in line with Reporting of studies Conducted using Observational Routinely collected health Data (RECORD) guidelines [14].

### Study design and data source

A retrospective population-based cohort study was conducted using data from The Health Improvement Network (THIN) database. THIN is a UK primary care database that contains anonymised electronic medical records (EMRs). As of May 2017, it includes longitudinal data on 16 million individuals (of which about 3 million are actively registered with a participating general practice at any one-time point) [15]. It covers approximately 6% of the actively registered population in the UK [15] and is representative of the UK general population in relation to age, sex, health conditions, major chronic illnesses, and mortality rates [16]. The validity and reliability of THIN data have been demonstrated for epidemiological research use [17], and has been used for studies of NAFLD [18, 19] and obesity [6].

### Study population

**Fig. 1:**
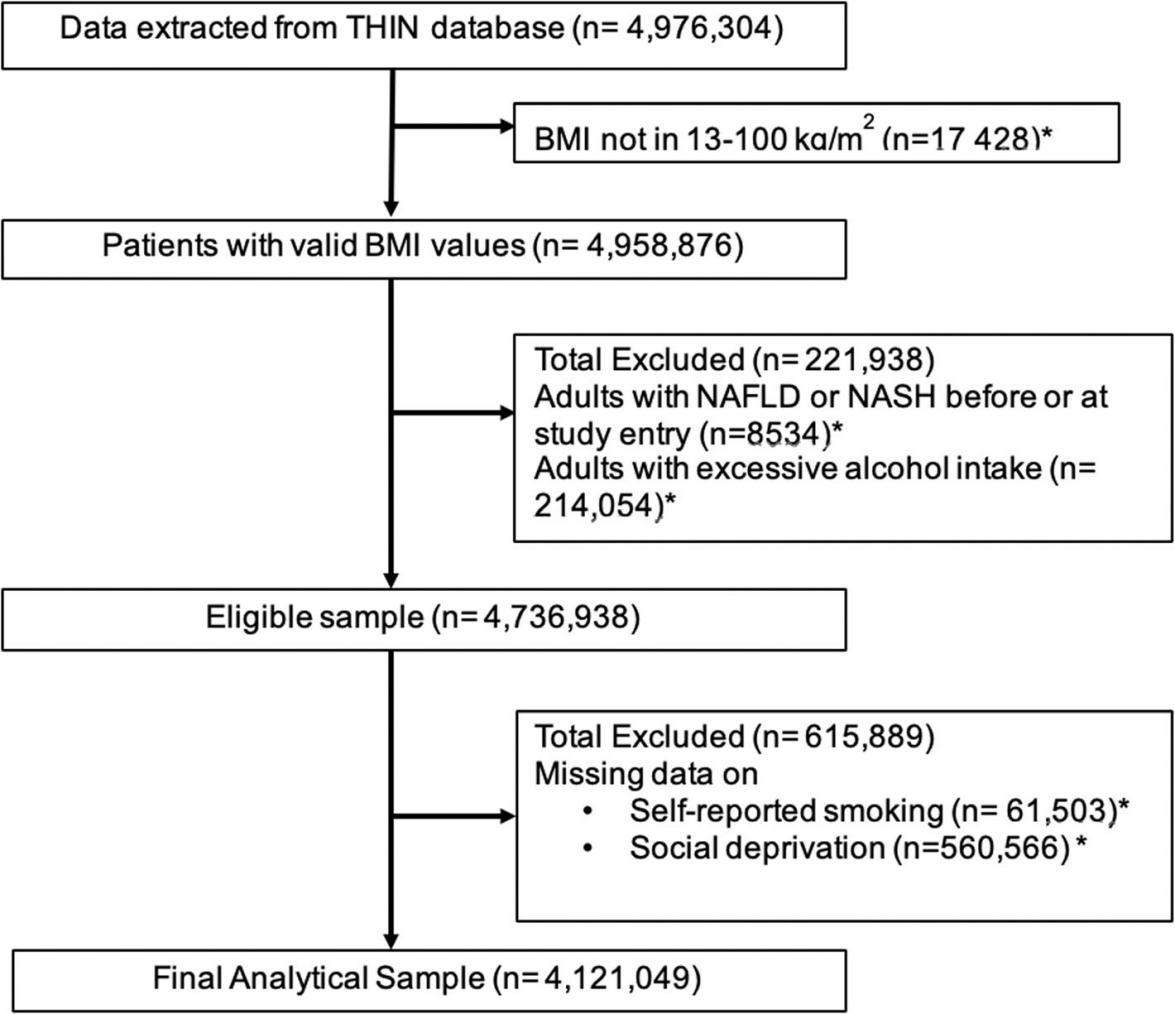
Flow diagram of study population

Only adults aged 18 years or over with body mass index data (BMI) data available after their registration with the practice but before the start of the study period (Fig. 1) in the THIN database between January 1995 to May 2017 were eligible to take part in the study. To ensure only incident outcomes were captured, participants were only included in the study after they had been registered with their practice for at least 1 year. This one-year period was chosen to limit the possibility that NAFLD diagnoses documented after the patient registration date represented pre-existing disease transferred from previous medical records rather than incident cases [20].

In addition, participants were also required to be from practices that had used the Vision computer system for at least 1 year and met the criteria for acceptable mortality reporting (AMR) (an indicator of quality for electronic data recording) for at least 1 year preceding their study entry date.

Adults with NAFLD before or at study entry were excluded. Patients with any documentation indicative of excessive alcohol intake at baseline were excluded to ensure cases of NAFLD that may have been inaccurately diagnosed were excluded. Selection of Read codes for excessive alcohol intake was based on a previous study which used Read codes related to hazardous drinking or conditions related to hazardous drinking [19] (Appendix 1).

Of this eligible sample, patients with implausible BMI values (below 13 kg/m^2^ or above 100 kg/m^2^) were excluded [15] and to permit adjustment for deprivation and smoking, patients with missing data were excluded.

### Study period

The study start date was 01 January 1995 and the study end date was 31 May 2017. The patient study entry date was the latest of the following: 12 months after patient registration date, 12 months after AMR or 12 months after Vision IT system implementation date. The study population was followed up until the first of the following events (i.e. exit date): the patient died; patient left practice, last data collection from practice or patient had a recorded diagnosis of NAFLD.

### Exposures

There is no standardised definition for metabolic health or accepted criteria for distinguishing between MHO and (metabolically unhealthy obese) MUO for research protocols or in clinical practice. In general, most studies use BMI > = 30 kg/m^2^ to define obesity and the absence of metabolic diseases such as diabetes, dyslipidaemia and hypertension to define “metabolically healthy” [21–23]. Therefore, in this study, individuals were grouped according to their BMI and whether they had any of those conditions [6].

#### Metabolic abnormalities

Diabetes and hypertension diagnoses were identified by Read code diagnoses at study entry date (Appendix 1). Recording of diabetes and hypertension in the primary care setting in the UK have been shown to be reliable [16] as they are conditions that are reported as part of the Quality Outcomes Framework which is a payment by performance scheme incentivising practices to keep an accurate register for these conditions [24]. Dyslipidaemia diagnosis was defined as those who were recorded to have been prescribed lipid-lowering medications using prescription codes or by laboratory measurements of elevated serum cholesterol or triglycerides or low-density lipoprotein-cholesterol (LDL-C) or low high-density lipoprotein-cholesterol (HDL-C) at baseline. The National Cholesterol Education Program (NCEP) Adult Treatment Panel (ATP) III classification of total and LDL-cholesterol were used to define high levels of total cholesterol as > 6.2 mmol/L (> 240 mg/dL) and high levels of LDL-C as > = 4.15 mmol/L (> = 160 mg/dL). The ATP III triglycerides and HDL-C cut-offs for metabolic syndrome was used to define elevated triglycerides as > = 1.7 mmol/L (> = 150 mg/dL) and low HDL-C as < 1.03 mmol/L (< 40 mg/dL) in men and < 1.29 mmol/L (< 50 mg/dL) in women [25].

Participants who had no recorded diabetes, hypertension or dyslipidaemia at study entry date (baseline status) but developed these metabolic abnormalities during the observation period were analysed according to their status at study entry.

#### BMI

BMI was categorised based on the World Health Organisation criteria: underweight (BMI of < 18.5 kg/m^2^), normal weight (BMI of 18.5 kg/m^2^ to < 25 kg/m^2^), overweight (BMI of 25 kg/m^2^ to < 30 kg/m^2^), and obese (BMI of ≥30 kg/m^2^) [26].

Baseline BMI was extracted from the dataset as the BMI recorded at time of patient registration or the first measurement when the practice was eligible to contribute towards the study (Fig. 1). This approach minimised the chance that the BMI was recorded due to particular clinical reasons but more likely to have been recorded for administrative purposes.

#### Subgroups characterised by metabolic health status and BMI

A metabolic abnormality score for each individual ranging from 0 to 3 was created by assigning one point each for the presence of a metabolic abnormality (diabetes, hypertension and dyslipidaemia), and categorised into 0, 1, 2 or more risk factors. These were then further stratified into 11 subgroups or phenotypes based on BMI.

### Outcome (dependent variable)

The primary outcome endpoint for analysis was a recorded diagnosis of NAFLD or NASH. Diagnoses of NAFLD and NASH were defined by Read codes (Appendix 1) [19].

### Covariates

Covariates considered for the analyses were age (continuous variable), sex (male or female), presence of hypothyroidism, self-reported smoking status (categorised as current; ex-smoker, never smoker) and social deprivation (categorical variable) on the patient’s record at study entry.

### Statistical analysis

#### Main analyses

The primary complete-case analyses included only patients with no missing or implausible data.

Incidence rates were calculated as the number of incident cases divided by person-years of follow-up. Incidence rate for NAFLD were reported by each phenotype based on BMI and metabolic status.

Cox proportional hazard models were used to assess the associations between each one of the eleven sub-groups and NAFLD to derive hazard ratios (HRs) and 95% confidence intervals (CIs). Normal weight and no metabolic abnormalities were selected as the reference category for all analyses. A hierarchical analytical approach was taken; firstly, unadjusted analyses were performed, which was subsequently adjusted for age at when BMI was recorded, sex, self-reported smoking status, and social deprivation. The proportional hazards assumption was tested by generating and visually inspecting log-log survival plots.

#### Sensitivity analyses

Since exclusions due to missing data may result in a selected sample, a sensitivity analysis was conducted where the available recording of Townsend score and smoking along with a missing category in the model were included. This analysis allowed for comparison of results with the results of the complete-case approach (primary analysis).

In addition, further analysis was conducted to check whether altering the definition of dyslipidaemia would alter the study conclusions. As previously noted, diabetes and hypertension are likely to be well recorded due to the QOF incentives scheme [24]. However, this is not the case for dyslipidaemia. A previous study classified dyslipidaemia by the prescription of lipid-modifying drugs only [6], therefore a sensitivity analysis was performed to assess the possibility of misclassification error due to our dyslipidaemia definition (Dyslipidaemia defined by drug lipid codes and laboratory measurements of cholesterol, triglycerides, LDL-C HDL-C vs dyslipidaemia defined by drug lipid codes only). These further analyses tested the robustness of the findings of the primary analyses.

Statistical significance was defined as *p* < 0.05. All data cleaning and statistical analyses was conducted in STATA version 15.0 (Stata Corp, College Station, TX).

### Ethics

The THIN organisation was granted permission to acquire and provide pseudonymised patient information to academics in 2003 by the National Health Service South-East Multi-centre Research Ethics Committee [27]. Authorization for this project were obtained from the University of Birmingham Scientific Review Committee (18THIN094).

## Results

4,976,304 adult patients with BMI values who were registered with practice that had Vision computer system and AMR at least for 1 year between January 1995 to May 2017 were extracted from the THIN database. Figure 1 shows the derivation of the final analytical sample of 4,121,049 patients (1,777,177 men and 2,343,872 women). Table 1 demonstrates the baseline characteristics of study participants by BMI category and metabolic health status.

**Table 1:** Baseline characteristics of study participants by body mass index category and metabolic health status (*n* = 4,121,049)

Characteristics	Overall	Underweight (***n*** = 110,084)	Normal Weight (n = 1,706,932)	Overweight (n = 1,377,896)	Metabolically healthy and Obese (***n*** = 466,571)	Metabolically Unhealthy^a^ and Obese (***n*** = 459,566)
**Age** median (IQR)	44.2 (30.4–60.6)	29.5 (26.7–56.1)	37.4 (26.7–56.1)	48.7 (34.8–63.4)	39.15 (29.7–50.1)	58.4 (48.3–67.9)
**Sex** n (%)						
Males	1,777,177 (43.1)	27,134 (24.5)	627,873 (36.8)	720,650 (52.3)	179,916 (38.6)	221,604 (48.2)
Females	2,343,872 (56.9)	82,950 (75.5)	1,079,059 (63.2)	657,246 (47.7)	286,655 (61.4)	237,962 (51.8)
**Townsend index** n (%)						
1	957,722 (23.2)	19,355 (17.6)	399,720 (23.4)	345,073 (25.0)	94,968(20.4)	98,606 (21.5)
2	868,163 (21.1)	19,187 (17.4)	351,685 (20.6)	306,703 (22.3)	93,059 (19.9)	97,529 (21.2)
3	894,788 (21.7)	23,498 (21.3)	367,703 (21.6)	297,443 (21.6)	104,192(22.3)	101,952 (22.2)
4	824,753 (20.0)	27,044 (24.6)	347,132 (20.3)	255,910 (18.6)	100,596(21.6)	94,071 (20.5)
5	575,623 (14.0)	21,000 (19.1)	240,692 (14.1)	172,767 (12.5)	73,756 (15.8)	67,408 (14.7)
Missing data	560,566	16,834	245,888	178,864	62,445	56,535
**Smoking status** n (%)						
Never smoker	2,310,052 (56.0)	62,861 (57.1)	989,362 (58.0)	755,361 (54.8)	262,833 (56.3)	239,635 (52.1)
Ex-smoker	875,978 (21.3)	12,920 (11.7)	290,992(17.0)	333,223(24.2)	94,049 (20.2)	144,794 (31.5)
Current smoker	935,019 (22.7)	34,303 (31.2)	426,578 (25.0)	289,312 (21.0)	109,689 (23.5)	75,137 (16.3)
Missing data	61,503	2640	24,862	18,869	10,042	5090
**Hypothyroidism** n (%)	165,477 (4.0)	3290 (3.0)	50,141 (2.9)	56,102 (4.1)	17,206 (3.7)	38,738 (8.4)
**BMI (kg/m**^**2**^**)** median (IQR)	25.7 (22.7–29.4)	17.6 (16.9–18.1)	22.4 (20.9–23.7)	27.1 (26–28.4)	33 (31.2–36.1)	33.4 (31.4–36.6)

^a^*Percentages exclude participants with missing data on Townsend score or Smoking Status*

### Missing data

Of the 4,736,938 eligible patients > = 18 years of age in the THIN database without a history of NAFLD or excessive alcohol intake at baseline, we excluded persons with missing data for smoking (61,503 [1.3%]) and social deprivation (560,566 [11.8%]). There was no missing data for age or sex. After these exclusions, there remained a final sample of 4,121,049 (87% of the eligible sample). The median follow-up period for participants was 4.7 years (IQR 1.9–8.7 years).

### Stability of metabolically healthy overweight and obese

Among initially overweight individuals with no metabolic abnormalities, approximately 1.2% developed diabetes, 6.8% developed hypertension and 17.3% dyslipidaemia (based on lipid prescription and dyslipidaemia READ codes) by the study end date. Among individuals who were initially MHO, approximately 3.6% developed diabetes, 9.8% developed hypertension and 21.7% dyslipidaemia by the study end date.

### Incidence rate of NAFLD

12,867 (0.3%) incident cases of NAFLD were identified during 23,254,181.4 person-years of follow up (incidence rate 0.55 per 1000 person-years). The incidence rate for NAFLD increased with each increasing BMI category and increasing number of metabolic abnormalities (Table 2) with the highest incidence rate (1.62 per 1000 person-years) seen in those who are obese with 2 or greater metabolic abnormalities.

**Table 2:** Incidence rates of NAFLD by each body size phenotype with and without metabolic abnormalities

Body size phenotype	Sample size	Incident Cases	Person-years	Incidence rate (per 1000 person-years)
Underweight, 0 metabolic abnormalities	94,189	23	418,897.5	0.05
Underweight, 1 metabolic abnormality	15,895	7	65,583.1	0.11
Normal weight, 0 metabolic abnormalities	1,367,321	882	7,137,510	0.12
Normal weight, 1 metabolic abnormality	223,270	377	1,287,137	0.29
Normal weight, ≥ 2 metabolic abnormalities	116,341	219	658,987.8	0.33
Overweight, 0 metabolic abnormalities	852,223	2112	4,926,580	0.43
Overweight, 1 metabolic abnormality	307,553	1437	1,885,388	0.76
Overweight, ≥ 2 metabolic abnormalities	218,120	1158	1,350,602	0.86
Obese, 0 metabolic abnormalities	466,571	2506	2,727,789	0.92
Obese, 1 metabolic abnormality	232,795	1909	1,412,798	1.35
Obese, ≥ 2 metabolic abnormalities	226,771	2237	1,382,909	1.62

### Association between metabolic health status and body size phenotype and NAFLD

**Fig. 2:**
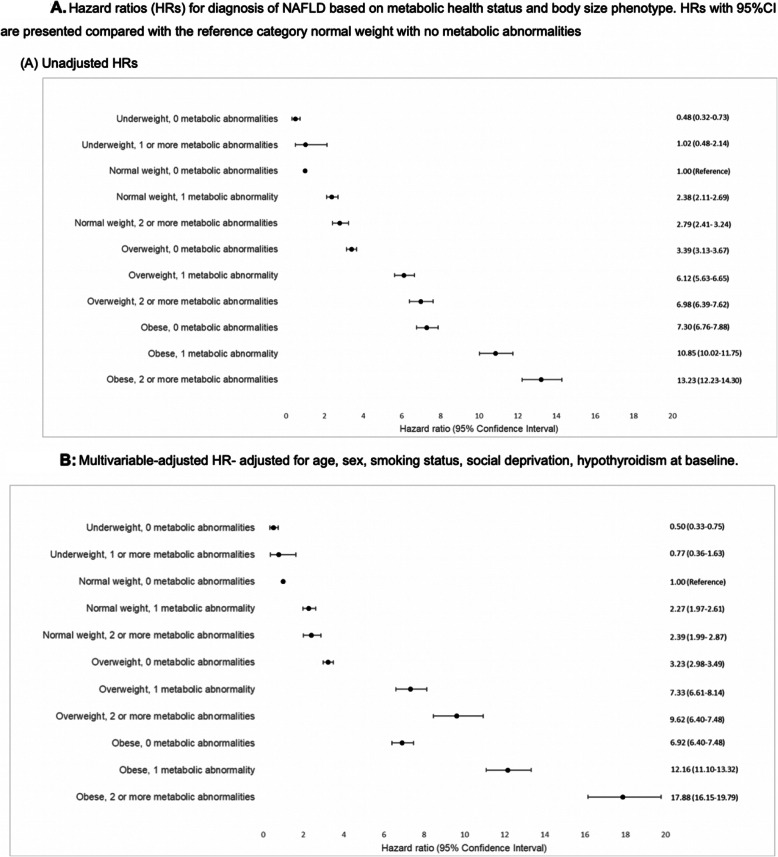
**a** Hazard ratios for diagnosis of NAFLD based on metabolic health status and body size phenotype. HRs with 95% CI are presented compared with the reference category, normal weight with no metabolic abnormalities. **b** Multivariable-adjusted HR – adjusted for age, sex, smoking status, social deprivation, hypothyroidism at baseline

Figure 2 presents the results of unadjusted and adjusted cox regression analyses undertaken to assess the risk of NAFLD among different metabolically defined body size phenotypes. Individuals who were overweight with no metabolic abnormalities were at nearly 3.2-fold higher risk of NAFLD (adjusted HR 3.23, 95%CI 2.98–3.49) than those who were normal weight with no metabolic abnormalities. This risk continued to rise with those individuals who were obese with no metabolic abnormalities having an adjusted HR of 6.92, 95%CI 6.40–7.48) compared to normal weight with no metabolic abnormalities (Fig. 2b).

Risk of NAFLD increased with each increasing BMI category (underweight, normal weight, overweight, obese) and within each BMI category risk increased with increasing number of metabolic abnormalities (Fig. 2b). Compared to individuals of normal weight with no metabolic abnormalities, those who were normal weight with metabolic abnormalities were associated with increased hazard of NAFLD (normal weight with one metabolic abnormality: adjusted HR 2.27, 95%CI 1.97–2.61), normal weight with two or more metabolic abnormalities: adjusted HR 2.39, 95%CI 1.99–2.87) (Fig. 2b). In addition, those who were underweight without metabolic abnormalities had a decreased risk of NAFLD (adjusted HR 0.50, 95%CI 0.33–0.75).

### Sensitivity analysis

Inclusion of those with missing information on social deprivation and smoking status in the regression model, did not significantly change the results (see Appendix 2, Table 1).

When dyslipidaemia was defined only by lipid-modifying drug prescriptions only, the size of the associations between body size phenotypes and metabolic status with NAFLD were slightly smaller but remained statistically significant (see Appendix 2, Table 2). The sensitivity analysis did not alter the conclusions of the study.

## Discussion

This study has three key findings. First, metabolically healthy individuals with overweight or obese were at increased risk of developing NAFLD in comparison with individuals of normal weight with no metabolic abnormalities. Secondly, individuals with normal weight who were metabolically unhealthy also had increased risk of NAFLD compared to their counterparts without metabolic abnormalities. Thirdly, the presence of metabolic abnormalities with obesity potentiates the risk of NAFLD. The NAFLD risk in metabolically unhealthy individuals with overweight and obesity was markedly greater than in their counterparts without metabolic abnormalities. While the association between obesity and metabolic health with NAFLD has been examined in previous Asian cohorts, this study provides the first evidence from a cohort study characterising this relationship in a European population.

The key findings of this study concur with results of previous studies, which are predominantly cross-sectional and cohort studies conducted in Asia. While the hazard ratio estimates were higher in our study in comparison to previous cohort studies conducted in China and Korea [7, 28], this may reflect differences in study populations and size. Neither of the two studies controlled for hypothyroidism, which may have biased results. In addition, the Korean retrospective cohort study was limited to only metabolically healthy participants [7], and in a relatively young population limiting the generalisability of their results. On the other hand, our study may have overestimated the effect sizes due to differentially higher surveillance in the obese population as well as the possibility that incidence cases of NAFLD likely represent a more severe phenotype as the majority of patients with NAFLD in the UK are undiagnosed.

The mechanisms underpinning the development of NAFLD are not fully understood. The traditional “two hit theory” may apply in regard to the role of obesity and metabolic abnormalities in the pathogenesis of NAFLD [29]. The “first hit” can be obesity/excess adiposity, leading to increased free fatty acid release and delivery to the liver resulting in hepatic steatosis, in turn sensitising the liver to the “second hit”. Adipose tissue also releases hormones and cytokines with pro-inflammatory effects such as leptin, interleukin-6 and tumour necrosis factor-alpha which may lead to the development of NAFLD. Furthermore, obesity increases the risk of obstructive sleep apnoea which is associated with NAFLD [30, 31]. Metabolic abnormalities may contribute to the “second hits”, such as inflammatory cytokines and oxidative stress, which result in further progression of NAFLD, to NASH and liver fibrosis/cirrhosis [32]. The findings of this study demonstrate that obesity independently increases the risk of NAFLD. Although this study cannot differentiate NASH from NAFLD, it has been previously suggested that excess adipose tissue may result in NASH and liver fibrosis without the “second hit” step [33]. Moreover, a study found that the expression of genes involved in inflammation were similarly modified in metabolically healthy and unhealthy obese individuals emphasising careful interpretation is needed when considering the MHO phenotype as harmless [34]. Indeed, lifestyle modification for even non-obese individuals with NAFLD has shown to significantly improve the degree of steatosis [35].

It has been proposed that the increased risk of adverse health outcomes reported for individuals with MHO indicates that this phenotype on a population-level is a transient state prior to further progression to the MUO state [36]. This is supported by the younger age and the high incidence of the metabolic abnormalities in this group. Studies with follow-up periods ranging from 11 to 20 years further support this contention [37–39].

Being a large primary care dataset, the THIN database has enabled access to millions of patient records in the UK, giving us unprecedented statistical power to evaluate the association between the MHO phenotype and the risk of developing incident NAFLD. To the best of our knowledge, this is the largest study to date and the first cohort study outside of Asia to look at this association. This large cohort allowed us to stratify individuals into subgroups according to the four main BMI categories and the number of metabolic abnormalities present, permitting a more detailed analysis than previously conducted studies with varying definitions of “metabolically healthy” patients. The present study used the number of concurrent metabolic abnormalities (0, 1, 2 or more) as being reflective of differing degrees of “metabolic health” in included patients. This in turn allowed us to examine whether there is a considerable impact on the risk of incident cases of NAFLD according to metabolic health-related co-morbidities. Additionally, a major strength of the study was being able to adjust for important baseline characteristics such as age, sex, smoking status, socio-economic deprivation and hypothyroidism which could confound the relationship between MHO and the risk of NAFLD. Sample bias was also limited by excluding individuals who had a history of alcohol excess at baseline. This ensured that cases of NAFLD that may have been diagnosed in error were excluded.

Considering only the baseline BMI in included patients could potentially have led to misclassification bias. However, as previously noted by Caleyachetty et al., due to the lack of successful and established weight loss interventions, individuals are likely to remain the same weight or gain weight compared to losing weight [6]. Therefore, it can be argued that the room for misclassification when adopting this baseline BMI approach for analysis is minimal and would likely lead to an underestimation as opposed to an overestimation of the risk for NAFLD. The impact of weight changes over time on NAFLD incidence would be useful to examine in another study, however, as THIN, a routinely collected primary care database lacks has relatively limited repeated assessment of BMI in all patients, another data source may be needed. Another limitation of the present study lies in the use of BMI to define obesity. BMI does not account for central adiposity, associated increase in waist circumference and visceral fat. Indeed, one difference between MHO and MUNO could be the proportion of visceral fat in these categories. The use of magnetic resonance imaging (MRI) to characterise body fat distribution in a study on adolescents has previously linked visceral fat with impaired glucose metabolism and hepatic fat accumulation [40]. A large-scale cross-sectional study has also explored the association between visceral fat and presence of NAFLD, discerning an elevated OR of 17.813 (95% CI 10.815–29.342) on comparing patients with normal and high level of visceral fat (> 100 cm^2^) [41]. However, given that the present study is carried out on a population-based primary care database, we were unable to obtain reliable data on visceral fat or waist circumference, hence had to use BMI.

It should also be noted that the observed associations in this study may not reflect the true relationship of metabolic status and obesity with NAFLD, as NAFLD may go undetected in the UK due to lack of systematic screening. The population prevalence estimated from studies where every participant underwent an ultrasound examination was much higher [42, 43] than the baseline prevalence of NAFLD in this study. This under-ascertainment of cases may be differential between those with and without obesity. For instance, those thought to be at risk, such as those with obesity or with other metabolic abnormalities, may be preferentially screened for NAFLD. Loomis et al. noted that the true risk of NAFLD in non-obese individuals could potentially be underestimated if the diagnosis of NAFLD is not being sought in those of normal weight. This may in turn overestimate the risk of NAFLD resulting from obesity [18]. Another limitation of the presented study is that use of READ codes could potentially fail to identify all subjects with NAFLD, and it is well recognised that NAFLD remains underdiagnosed in the community. Despite this, a primary care database such as THIN that provides a recorded diagnosis of NAFLD appears to be the most appropriate method to obtain data on incident NAFLD for a large population-based epidemiological study in the UK. This study provides a useful estimate of recorded NAFLD in clinical practice in UK according to subgroups based on BMI and metabolic health status, reflecting “real-life” care.

However, the main results of this study are likely robust and externally valid due to the following reasons. The finding that MHO individuals are at higher risk of NAFLD compared to individuals of normal weight with no metabolic abnormalities is consistent with the findings of all cross-sectional and cohort studies from other settings. A large cohort study conducted in a metabolically healthy Korean population who had annual ultrasound examinations reported a 4-fold difference in NAFLD incidence rate between normal weight and obese individuals (incidence rate 20.3 per 1000 person-years in normal weight group vs incidence rate 85.9 per 1000 person-years in obese group) [7]. In addition, the unadjusted and adjusted analyses in this study examining the risk of NAFLD among individuals in the different obese subgroups resulted in large HRs above 6. The magnitude of the results in this study may suggest causality as any bias or residual confounding is unlikely to greatly attenuate the strong association observed.

## Conclusions

In conclusion, individuals with MHO are at greater risk of developing NAFLD compared to metabolically healthy normal weight individuals. Additionally, the incidence of NAFLD increased with the increasing number of metabolic abnormalities in overweight and obese patients. These findings support that MHO phenotype is a fallacy and using the term may mislead individuals to believe obesity can be “healthy”. This study has also highlighted that individuals considered to be of normal weight can have metabolic abnormalities and can be at higher risk of NAFLD. Therefore, clinicians should be aware that NAFLD can occur in this subset of normal weight individuals and may need to counsel them on management to help prevent progression to adverse liver and cardiometabolic outcomes.

## Supplementary information

**Additional file 1:Appendix 1.** Read Codes. **Appendix 2.** Sensitivity Analyses **Table S1.Table S2.** Development of NAFLD by body size phenotype and metabolic status using metabolic status derived from diagnostic codes for diabetes and hypertension and prescription records of lipid-modifying drugs only.

## Data Availability

Patient data not available for sharing.

## Abbreviations

NAFLD: Non-alcoholic fatty liver disease
MHO: Metabolically healthy obese
THIN: The Health Improvement Network
MUNO: Metabolically unhealthy non-obese
BMI: Body mass index
