# Supplementary material for: Impact of obesity and metabolic health status in the development of non-alcoholic fatty liver disease (NAFLD): A United Kingdom population-based cohort study using the health improvement network (THIN)
Source: BMC Endocr Disord. 2020 Jun 30;20:96. doi: 10.1186/s12902-020-00582-9 (PMC7325099; doi:10.1186/s12902-020-00582-9)
Supplement: Supplementary file 1 — Additional file 1:Appendix 1. Read Codes. Appendix 2. Sensitivity Analyses Table S1.Table S2. Development of NAFLD by body size phenotype and metabolic status using metabolic status derived from diagnostic codes for diabetes and hypertension and prescription records of lipid-modifying drugs only. [file 12902_2020_582_MOESM1_ESM.docx]

**APPENDIX 1**: **Read Codes**

**Read codes for Diabetes**

| Diabetes | READ code | Description |
| --- | --- | --- |
|  | C10.00 | Diabetes mellitus |
|  | C100.00 | Diabetes mellitus with no mention of complication |
|  | C100000 | Diabetes mellitus, juvenile type, no mention of complication |
|  | C100011 | Insulin dependent diabetes mellitus |
|  | C100100 | Diabetes mellitus, adult onset, no mention of complication |
|  | C100111 | Maturity onset diabetes |
|  | C100112 | Non-insulin dependent diabetes mellitus |
|  | C100z00 | Diabetes mellitus NOS with no mention of complication |
|  | C101.00 | Diabetes mellitus with ketoacidosis |
|  | C101000 | Diabetes mellitus, juvenile type, with ketoacidosis |
|  | C101100 | Diabetes mellitus, adult onset, with ketoacidosis |
|  | C101y00 | Other specified diabetes mellitus with ketoacidosis |
|  | C101z00 | Diabetes mellitus NOS with ketoacidosis |
|  | C102.00 | Diabetes mellitus with hyperosmolar coma |
|  | C102000 | Diabetes mellitus, juvenile type, with hyperosmolar coma |
|  | C102100 | Diabetes mellitus, adult onset, with hyperosmolar coma |
|  | C102z00 | Diabetes mellitus NOS with hyperosmolar coma |
|  | C103.00 | Diabetes mellitus with ketoacidotic coma |
|  | C103000 | Diabetes mellitus, juvenile type, with ketoacidotic coma |
|  | C103100 | Diabetes mellitus, adult onset, with ketoacidotic coma |
|  | C103y00 | Other specified diabetes mellitus with coma |
|  | C103z00 | Diabetes mellitus NOS with ketoacidotic coma |
|  | C104.00 | Diabetes mellitus with renal manifestation |
|  | C104.11 | Diabetic nephropathy |
|  | C104000 | Diabetes mellitus, juvenile type, with renal manifestation |
|  | C104100 | Diabetes mellitus, adult onset, with renal manifestation |
|  | C104y00 | Other specified diabetes mellitus with renal complications |
|  | C104z00 | Diabetes mellitus with nephropathy NOS |
|  | C105.00 | Diabetes mellitus with ophthalmic manifestation |
|  | C105000 | Diabetes mellitus, juvenile type, + ophthalmic manifestation |
|  | C105100 | Diabetes mellitus, adult onset, + ophthalmic manifestation |
|  | C105y00 | Other specified diabetes mellitus with ophthalmic complication |
|  | C105z00 | Diabetes mellitus NOS with ophthalmic manifestation |
|  | C106.00 | Diabetes mellitus with neurological manifestation |
|  | C106.11 | Diabetic amyotrophy |
|  | C106.12 | Diabetes mellitus with neuropathy |
|  | C106.13 | Diabetes mellitus with polyneuropathy |
|  | C106000 | Diabetes mellitus, juvenile, + neurological manifestation |
|  | C106100 | Diabetes mellitus, adult onset, + neurological manifestation |
|  | C106y00 | Other specified diabetes mellitus with neurological complications |
|  | C106z00 | Diabetes mellitus NOS with neurological manifestation |
|  | C108.00 | Insulin dependent diabetes mellitus |
|  | C108.11 | IDDM-Insulin dependent diabetes mellitus |
|  | C108.12 | Type 1 diabetes mellitus |
|  | C108.13 | Type I diabetes mellitus |
|  | C108000 | Insulin-dependent diabetes mellitus with renal complications |
|  | C108011 | Type I diabetes mellitus with renal complications |
|  | C108012 | Type 1 diabetes mellitus with renal complications |
|  | C108100 | Insulin-dependent diabetes mellitus with ophthalmic complications |
|  | C108111 | Type I diabetes mellitus with ophthalmic complications |
|  | C108112 | Type 1 diabetes mellitus with ophthalmic complications |
|  | C108200 | Insulin-dependent diabetes mellitus with neurological complications |
|  | C108211 | Type I diabetes mellitus with neurological complications |
|  | C108212 | Type 1 diabetes mellitus with neurological complications |
|  | C108300 | Insulin dependent diabetes mellitus with multiple complication |
|  | C108311 | Type I diabetes mellitus with multiple complications |
|  | C108312 | Type 1 diabetes mellitus with multiple complications |
|  | C108400 | Unstable insulin dependent diabetes mellitus |
|  | C108411 | Unstable type I diabetes mellitus |
|  | C108412 | Unstable type 1 diabetes mellitus |
|  | C108500 | Insulin dependent diabetes mellitus with ulcer |
|  | C108511 | Type I diabetes mellitus with ulcer |
|  | C108512 | Type 1 diabetes mellitus with ulcer |
|  | C108700 | Insulin dependent diabetes mellitus with retinopathy |
|  | C108711 | Type I diabetes mellitus with retinopathy |
|  | C108712 | Type 1 diabetes mellitus with retinopathy |
|  | C108800 | Insulin dependent diabetes mellitus - poor control |
|  | C108811 | Type I diabetes mellitus - poor control |
|  | C108812 | Type 1 diabetes mellitus - poor control |
|  | C108900 | Insulin dependent diabetes maturity onset |
|  | C108911 | Type I diabetes mellitus maturity onset |
|  | C108912 | Type 1 diabetes mellitus maturity onset |
|  | C108A00 | Insulin-dependent diabetes without complication |
|  | C108A11 | Type I diabetes mellitus without complication |
|  | C108A12 | Type 1 diabetes mellitus without complication |
|  | C108B00 | Insulin dependent diabetes mellitus with mononeuropathy |
|  | C108B11 | Type I diabetes mellitus with mononeuropathy |
|  | C108B12 | Type 1 diabetes mellitus with mononeuropathy |
|  | C108C00 | Insulin dependent diabetes mellitus with polyneuropathy |
|  | C108C11 | Type I diabetes mellitus with polyneuropathy |
|  | C108C12 | Type 1 diabetes mellitus with polyneuropathy |
|  | C108D00 | Insulin dependent diabetes mellitus with nephropathy |
|  | C108D11 | Type I diabetes mellitus with nephropathy |
|  | C108D12 | Type 1 diabetes mellitus with nephropathy |
|  | C108E00 | Insulin dependent diabetes mellitus with hypoglycemic coma |
|  | C108E11 | Type I diabetes mellitus with hypoglycemic coma |
|  | C108E12 | Type 1 diabetes mellitus with hypoglycemic coma |
|  | C108F00 | Insulin dependent diabetes mellitus with diabetic cataract |
|  | C108F11 | Type I diabetes mellitus with diabetic cataract |
|  | C108F12 | Type 1 diabetes mellitus with diabetic cataract |
|  | C108G00 | Insulin dependent diabetes mellitus with peripheral angiopathy |
|  | C108G11 | Type I diabetes mellitus with peripheral angiopathy |
|  | C108G12 | Type 1 diabetes mellitus with peripheral angiopathy |
|  | C108H00 | Insulin dependent diabetes mellitus with arthropathy |
|  | C108H11 | Type I diabetes mellitus with arthropathy |
|  | C108H12 | Type 1 diabetes mellitus with arthropathy |
|  | C108J00 | Insulin dependent diabetes mellitus with neuropathic arthropathy |
|  | C108J11 | Type I diabetes mellitus with neuropathic arthropathy |
|  | C108J12 | Type 1 diabetes mellitus with neuropathic arthropathy |
|  | C108y00 | Other specified diabetes mellitus with multiple complications |
|  | C108z00 | Unspecified diabetes mellitus with multiple complications |
|  | C109.00 | Non-insulin dependent diabetes mellitus |
|  | C109.11 | NIDDM - Non-insulin dependent diabetes mellitus |
|  | C109.12 | Type 2 diabetes mellitus |
|  | C109.13 | Type II diabetes mellitus |
|  | C109000 | Non-insulin-dependent diabetes mellitus with renal complications |
|  | C109011 | Type II diabetes mellitus with renal complications |
|  | C109012 | Type 2 diabetes mellitus with renal complications |
|  | C109100 | Non-insulin-dependent diabetes mellitus with ophthalmic complications |
|  | C109111 | Type II diabetes mellitus with ophthalmic complications |
|  | C109112 | Type 2 diabetes mellitus with ophthalmic complications |
|  | C109200 | Non-insulin-dependent diabetes mellitus with neuro complications |
|  | C109211 | Type II diabetes mellitus with neurological complications |
|  | C109212 | Type 2 diabetes mellitus with neurological complications |
|  | C109300 | Non-insulin-dependent diabetes mellitus with multiple complications |
|  | C109311 | Type II diabetes mellitus with multiple complications |
|  | C109312 | Type 2 diabetes mellitus with multiple complications |
|  | C109400 | Non-insulin dependent diabetes mellitus with ulcer |
|  | C109411 | Type II diabetes mellitus with ulcer |
|  | C109412 | Type 2 diabetes mellitus with ulcer |
|  | C109600 | Non-insulin-dependent diabetes mellitus with retinopathy |
|  | C109611 | Type II diabetes mellitus with retinopathy |
|  | C109612 | Type 2 diabetes mellitus with retinopathy |
|  | C109700 | Non-insulin dependent diabetes mellitus - poor control |
|  | C109711 | Type II diabetes mellitus - poor control |
|  | C109712 | Type 2 diabetes mellitus - poor control |
|  | C109800 | Reaven's syndrome |
|  | C109900 | Non-insulin-dependent diabetes mellitus without complication |
|  | C109911 | Type II diabetes mellitus without complication |
|  | C109912 | Type 2 diabetes mellitus without complication |
|  | C109A00 | Non-insulin dependent diabetes mellitus with mononeuropathy |
|  | C109A11 | Type II diabetes mellitus with mononeuropathy |
|  | C109A12 | Type 2 diabetes mellitus with mononeuropathy |
|  | C109B00 | Non-insulin dependent diabetes mellitus with polyneuropathy |
|  | C109B11 | Type II diabetes mellitus with polyneuropathy |
|  | C109B12 | Type 2 diabetes mellitus with polyneuropathy |
|  | C109C00 | Non-insulin dependent diabetes mellitus with nephropathy |
|  | C109C11 | Type II diabetes mellitus with nephropathy |
|  | C109C12 | Type 2 diabetes mellitus with nephropathy |
|  | C109D00 | Non-insulin dependent diabetes mellitus with hypoglycemic coma |
|  | C109D11 | Type II diabetes mellitus with hypoglycemic coma |
|  | C109D12 | Type 2 diabetes mellitus with hypoglycemic coma |
|  | C109E00 | Non-insulin depend diabetes mellitus with diabetic cataract |
|  | C109E11 | Type II diabetes mellitus with diabetic cataract |
|  | C109E12 | Type 2 diabetes mellitus with diabetic cataract |
|  | C109G00 | Non-insulin dependent diabetes mellitus with arthropathy |
|  | C109G11 | Type II diabetes mellitus with arthropathy |
|  | C109G12 | Type 2 diabetes mellitus with arthropathy |
|  | C109H00 | Non-insulin dependent d m with neuropathic arthropathy |
|  | C109H11 | Type II diabetes mellitus with neuropathic arthropathy |
|  | C109H12 | Type 2 diabetes mellitus with neuropathic arthropathy |
|  | C109J00 | Insulin treated Type 2 diabetes mellitus |
|  | C109J11 | Insulin treated non-insulin dependent diabetes mellitus |
|  | C109J12 | Insulin treated Type II diabetes mellitus |
|  | C109K00 | Hyperosmolar non-ketotic state in type 2 diabetes mellitus |
|  | C10A.00 | Malnutrition-related diabetes mellitus |
|  | C10A000 | Malnutrition-related diabetes mellitus with coma |
|  | C10A100 | Malnutrition-related diabetes mellitus with ketoacidosis |
|  | C10A200 | Malnutrition-related diabetes mellitus with renal complication |
|  | C10A300 | Malnutrition-related diabetes mellitus with ophthalmic complication |
|  | C10A400 | Malnutrition-related diabetes mellitus with neuro complications |
|  | C10A600 | Malnutrition-related diabetes mellitus with multiple complications |
|  | C10A700 | Malnutrition-related diabetes mellitus without complications |
|  | C10AW00 | Malnutrition-related diabetes mellitus with unspecified complications |
|  | C10AX00 | Malnutrition-related diabetes mellitus with other spec complications |
|  | C10B.00 | Diabetes mellitus induced by steroids |
|  | C10B000 | Steroid induced diabetes mellitus without complication |
|  | C10C.00 | Diabetes mellitus autosomal dominant |
|  | C10C.11 | Maturity onset diabetes in youth |
|  | C10C.12 | Maturity onset diabetes in youth type 1 |
|  | C10D.00 | Diabetes mellitus autosomal dominant type 2 |
|  | C10D.11 | Maturity onset diabetes in youth type 2 |
|  | C10E.00 | Type 1 diabetes mellitus |
|  | C10E.11 | Type I diabetes mellitus |
|  | C10E.12 | Insulin dependent diabetes mellitus |
|  | C10E000 | Type 1 diabetes mellitus with renal complications |
|  | C10E011 | Type I diabetes mellitus with renal complications |
|  | C10E012 | Insulin-dependent diabetes mellitus with renal complications |
|  | C10E100 | Type 1 diabetes mellitus with ophthalmic complications |
|  | C10E111 | Type I diabetes mellitus with ophthalmic complications |
|  | C10E112 | Insulin-dependent diabetes mellitus with ophthalmic complications |
|  | C10E200 | Type 1 diabetes mellitus with neurological complications |
|  | C10E211 | Type I diabetes mellitus with neurological complications |
|  | C10E212 | Insulin-dependent diabetes mellitus with neurological complications |
|  | C10E300 | Type 1 diabetes mellitus with multiple complications |
|  | C10E311 | Type I diabetes mellitus with multiple complications |
|  | C10E312 | Insulin dependent diabetes mellitus with multiple complication |
|  | C10E400 | Unstable type 1 diabetes mellitus |
|  | C10E411 | Unstable type I diabetes mellitus |
|  | C10E412 | Unstable insulin dependent diabetes mellitus |
|  | C10E500 | Type 1 diabetes mellitus with ulcer |
|  | C10E511 | Type I diabetes mellitus with ulcer |
|  | C10E512 | Insulin dependent diabetes mellitus with ulcer |
|  | C10E700 | Type 1 diabetes mellitus with retinopathy |
|  | C10E711 | Type I diabetes mellitus with retinopathy |
|  | C10E712 | Insulin dependent diabetes mellitus with retinopathy |
|  | C10E800 | Type 1 diabetes mellitus - poor control |
|  | C10E811 | Type I diabetes mellitus - poor control |
|  | C10E812 | Insulin dependent diabetes mellitus - poor control |
|  | C10E900 | Type 1 diabetes mellitus maturity onset |
|  | C10E911 | Type I diabetes mellitus maturity onset |
|  | C10E912 | Insulin dependent diabetes maturity onset |
|  | C10EA00 | Type 1 diabetes mellitus without complication |
|  | C10EA11 | Type I diabetes mellitus without complication |
|  | C10EA12 | Insulin-dependent diabetes without complication |
|  | C10EB00 | Type 1 diabetes mellitus with mononeuropathy |
|  | C10EB11 | Type I diabetes mellitus with mononeuropathy |
|  | C10EB12 | Insulin dependent diabetes mellitus with mononeuropathy |
|  | C10EC00 | Type 1 diabetes mellitus with polyneuropathy |
|  | C10EC11 | Type I diabetes mellitus with polyneuropathy |
|  | C10EC12 | Insulin dependent diabetes mellitus with polyneuropathy |
|  | C10ED00 | Type 1 diabetes mellitus with nephropathy |
|  | C10ED11 | Type I diabetes mellitus with nephropathy |
|  | C10ED12 | Insulin dependent diabetes mellitus with nephropathy |
|  | C10EE00 | Type 1 diabetes mellitus with hypoglycemic coma |
|  | C10EE11 | Type I diabetes mellitus with hypoglycemic coma |
|  | C10EE12 | Insulin dependent diabetes mellitus with hypoglycemic coma |
|  | C10EF00 | Type 1 diabetes mellitus with diabetic cataract |
|  | C10EF11 | Type I diabetes mellitus with diabetic cataract |
|  | C10EF12 | Insulin dependent diabetes mellitus with diabetic cataract |
|  | C10EH00 | Type 1 diabetes mellitus with arthropathy |
|  | C10EH11 | Type I diabetes mellitus with arthropathy |
|  | C10EH12 | Insulin dependent diabetes mellitus with arthropathy |
|  | C10EJ00 | Type 1 diabetes mellitus with neuropathic arthropathy |
|  | C10EJ11 | Type I diabetes mellitus with neuropathic arthropathy |
|  | C10EJ12 | Insulin dependent diabetes mellitus with neuropathic arthropathy |
|  | C10EK00 | Type 1 diabetes mellitus with persistent proteinuria |
|  | C10EK11 | Type I diabetes mellitus with persistent proteinuria |
|  | C10EL00 | Type 1 diabetes mellitus with persistent microalbuminuria |
|  | C10EL11 | Type I diabetes mellitus with persistent microalbuminuria |
|  | C10EM00 | Type 1 diabetes mellitus with ketoacidosis |
|  | C10EM11 | Type I diabetes mellitus with ketoacidosis |
|  | C10EN00 | Type 1 diabetes mellitus with ketoacidotic coma |
|  | C10EN11 | Type I diabetes mellitus with ketoacidotic coma |
|  | C10EP00 | Type 1 diabetes mellitus with exudative maculopathy |
|  | C10EP11 | Type I diabetes mellitus with exudative maculopathy |
|  | C10EQ00 | Type 1 diabetes mellitus with gastroparesis |
|  | C10EQ11 | Type I diabetes mellitus with gastroparesis |
|  | C10ER00 | Latent autoimmune diabetes mellitus in adult |
|  | C10F.00 | Type 2 diabetes mellitus |
|  | C10F.11 | Type II diabetes mellitus |
|  | C10F000 | Type 2 diabetes mellitus with renal complications |
|  | C10F011 | Type II diabetes mellitus with renal complications |
|  | C10F100 | Type 2 diabetes mellitus with ophthalmic complications |
|  | C10F111 | Type II diabetes mellitus with ophthalmic complications |
|  | C10F200 | Type 2 diabetes mellitus with neurological complications |
|  | C10F211 | Type II diabetes mellitus with neurological complications |
|  | C10F300 | Type 2 diabetes mellitus with multiple complications |
|  | C10F311 | Type II diabetes mellitus with multiple complications |
|  | C10F400 | Type 2 diabetes mellitus with ulcer |
|  | C10F411 | Type II diabetes mellitus with ulcer |
|  | C10F600 | Type 2 diabetes mellitus with retinopathy |
|  | C10F611 | Type II diabetes mellitus with retinopathy |
|  | C10F700 | Type 2 diabetes mellitus - poor control |
|  | C10F711 | Type II diabetes mellitus - poor control |
|  | C10F800 | Reaven's syndrome |
|  | C10F811 | Metabolic syndrome X |
|  | C10F900 | Type 2 diabetes mellitus without complication |
|  | C10F911 | Type II diabetes mellitus without complication |
|  | C10FA00 | Type 2 diabetes mellitus with mononeuropathy |
|  | C10FA11 | Type II diabetes mellitus with mononeuropathy |
|  | C10FB00 | Type 2 diabetes mellitus with polyneuropathy |
|  | C10FB11 | Type II diabetes mellitus with polyneuropathy |
|  | C10FC00 | Type 2 diabetes mellitus with nephropathy |
|  | C10FC11 | Type II diabetes mellitus with nephropathy |
|  | C10FD00 | Type 2 diabetes mellitus with hypoglycemic coma |
|  | C10FD11 | Type II diabetes mellitus with hypoglycemic coma |
|  | C10FE00 | Type 2 diabetes mellitus with diabetic cataract |
|  | C10FE11 | Type II diabetes mellitus with diabetic cataract |
|  | C10FG00 | Type 2 diabetes mellitus with arthropathy |
|  | C10FG11 | Type II diabetes mellitus with arthropathy |
|  | C10FH00 | Type 2 diabetes mellitus with neuropathic arthropathy |
|  | C10FH11 | Type II diabetes mellitus with neuropathic arthropathy |
|  | C10FJ00 | Insulin treated Type 2 diabetes mellitus |
|  | C10FJ11 | Insulin treated Type II diabetes mellitus |
|  | C10FK00 | Hyperosmolar non-ketotic state in type 2 diabetes mellitus |
|  | C10FK11 | Hyperosmolar non-ketotic state in type II diabetes mellitus |
|  | C10FL00 | Type 2 diabetes mellitus with persistent proteinuria |
|  | C10FL11 | Type II diabetes mellitus with persistent proteinuria |
|  | C10FM00 | Type 2 diabetes mellitus with persistent microalbuminuria |
|  | C10FM11 | Type II diabetes mellitus with persistent microalbuminuria |
|  | C10FN00 | Type 2 diabetes mellitus |

**Read codes for Hypertension**

| Hypertension | READ code | Description |
| --- | --- | --- |
|  | G2...00 | Hypertensive disease |
|  | G2...11 | BP - hypertensive disease |
|  | G20..00 | Essential hypertension |
|  | G20..11 | High blood pressure |
|  | G20..12 | Primary hypertension |
|  | G200.00 | Malignant essential hypertension |
|  | G201.00 | Benign essential hypertension |
|  | G202.00 | Systolic hypertension |
|  | G203.00 | Diastolic hypertension |
|  | G20z.00 | Essential hypertension NOS |
|  | G20z.11 | Hypertension NOS |
|  | G24..00 | Secondary hypertension |
|  | G240.00 | Secondary malignant hypertension |
|  | G240000 | Secondary malignant renovascular hypertension |
|  | G240z00 | Secondary malignant hypertension NOS |
|  | G241.00 | Secondary benign hypertension |
|  | G241000 | Secondary benign renovascular hypertension |
|  | G241z00 | Secondary benign hypertension NOS |
|  | G244.00 | Hypertension secondary to endocrine disorders |
|  | G24z.00 | Secondary hypertension NOS |
|  | G24z000 | Secondary renovascular hypertension NOS |
|  | G24z100 | Hypertension secondary to drug |
|  | G24zz00 | Secondary hypertension NOS |
|  | G25..00 | Stage 1 hypertension (NICE - National Institute for Health and Care Excellence 2011) |
|  | G25..11 | Stage 1 hypertension |
|  | G250.00 | Stage 1 hypertension (NICE 2011) without evidence of end organ damage |
|  | G251.00 | Stage 1 hypertension (NICE 2011) with evidence of end organ damage |
|  | G26..00 | Severe hypertension (NICE 2011) |
|  | G26..11 | Severe hypertension |
|  | G27..00 | Hypertension resistant to drug therapy |
|  | G28..00 | Stage 2 hypertension (NICE 2011) |
|  | Gyu2000 | [X]Other secondary hypertension |
|  | Gyu2100 | [X]Hypertension secondary to other renal disorders |

**Drug codes for Lipid-modifying drugs**

| Lipid-modifying drugs | Drug Code | Description |
| --- | --- | --- |
|  | 81048998 | Atorvastatin 20mg chewable tablets sugar free |
|  | 81051998 | Atorvastatin 10mg chewable tablets sugar free |
|  | 83099998 | Simvastatin 40mg/5ml oral suspension sugar free |
|  | 82655998 | Nicotinic acid & laropiprant 1g+20mg tablets |
|  | 83030998 | Simvastatin 80mg tablets |
|  | 81050998 | Atorvastatin 10mg chewable tablets sugar free |
|  | 84268998 | Colesevelam 625mg tablets |
|  | 84267998 | Colesevelam 625mg tablets |
|  | 83594998 | Nicotinic acid 1g / laropiprant 20mg modified-release tablets |
|  | 79254979 | Simvastatin 20mg/5ml oral suspension sugar free |
|  | 83188998 | Bezafibrate 200mg tablets |
|  | 83187998 | Bezafibrate 400mg modified-release tablets |
|  | 81049998 | Atorvastatin 20mg chewable tablets sugar free |
|  | 82141978 | Eicosapentaenoic acid 460mg / Docosahexaenoic acid 380mg capsules |
|  | 87853998 | Nicotinic acid 1g modified-release tablets |
|  | 87852998 | Nicotinic acid 500mg modified release tablets |
|  | 89154996 | Cerivastatin 300microgram tablets |
|  | 86791998 | Simvastatin 80mg / Ezetimibe 10mg tablets |
|  | 87854998 | Nicotinic acid 750mg modified-release tablets |
|  | 89153996 | Cerivastatin sodium 300mcg tablets |
|  | 88298997 | Fenofibrate micronised 267mg capsules |
|  | 88534998 | Rosuvastatin 10mg tablets |
|  | 86794998 | Simvastatin 80mg / Ezetimibe 10mg tablets |
|  | 86510979 | Ispaghula husk 3.5g sugar free granules |
|  | 87025998 | Bezafibrate 400mg modified-release tablets |
|  | 87418998 | Simvastatin 10mg tablets |
|  | 87918998 | Simvastatin 10mg tablets |
|  | 89401998 | Bezafibrate 400mg modified-release tablets |
|  | 87917998 | Simvastatin 20mg tablets |
|  | 89089998 | Bezafibrate 400mg modified release tablets |
|  | 87373998 | Simvastatin 10mg tablets |
|  | 87760998 | Colestipol 5g granules sachets sugar free |
|  | 86798998 | Simvastatin 20mg / Ezetimibe 10mg tablets |
|  | 88297996 | Fenofibrate micronised 267mg capsules |
|  | 87848998 | Nicotinic acid pack |
|  | 86796998 | Simvastatin 40mg / Ezetimibe 10mg tablets |
|  | 87849998 | Nicotinic acid 375mg + 500mg + 750mg modified-release tablet |
|  | 87850998 | Nicotinic acid 1g modified release tablets |
|  | 87851998 | Nicotinic acid 750mg modified release tablets |
|  | 86797998 | Simvastatin 20mg / Ezetimibe 10mg tablets |
|  | 87916998 | Simvastatin 40mg tablets |
|  | 89306996 | Atorvastatin 40mg tablets |
|  | 89311998 | Atorvastatin 10mg tablets |
|  | 89617998 | Ispaghula husk 3.5g sugar free granules |
|  | 89154997 | Cerivastatin 200microgram tablets |
|  | 86795998 | Simvastatin 40mg / Ezetimibe 10mg tablets |
|  | 89311997 | Atorvastatin 20mg tablets |
|  | 89306998 | Atorvastatin 10mg tablets |
|  | 89311996 | Atorvastatin 40mg tablets |
|  | 88298996 | Fenofibrate micronised 200mg capsules |
|  | 86788998 | Simvastatin 40mg / Ezetimibe 10mg tablets |
|  | 86789998 | Simvastatin 20mg / Ezetimibe 10mg tablets |
|  | 89153998 | Cerivastatin sodium 100mcg tablets |
|  | 86787998 | Simvastatin 80mg / Ezetimibe 10mg tablets |
|  | 89154998 | Cerivastatin 100microgram tablets |
|  | 88297998 | Fenofibrate micronised 67mg capsules |
|  | 86467998 | Rosuvastatin 5mg tablets |
|  | 89285979 | Nicotinic acid 500mg modified release tablets |
|  | 89800998 | Eicosapentaenoic acid 460mg / Docosahexaenoic acid 380mg capsules |
|  | 89284979 | Nicotinic acid 750mg modified release tablets |
|  | 88298998 | Fenofibrate micronised 67mg capsules |
|  | 88297997 | Fenofibrate micronised 200mg capsules |
|  | 89306997 | Atorvastatin 20mg tablets |
|  | 87855998 | Nicotinic acid 500mg modified-release tablets |
|  | 89283979 | Nicotinic acid 1g modified release tablets |
|  | 89153997 | Cerivastatin sodium 200mcg tablets |
|  | 86468998 | Rosuvastatin 5mg tablets |
|  | 92447998 | Cerivastatin sodium 400mcg tablets |
|  | 90973998 | Rosuvastatin 20mg tablets |
|  | 93619998 | Simvastatin 10mg tablets |
|  | 92408998 | Rosuvastatin 20mg tablets |
|  | 93620996 | Simvastatin 40mg tablets |
|  | 92448997 | Cerivastatin 800microgram tablets |
|  | 92410998 | Rosuvastatin 40mg tablets |
|  | 93620997 | Simvastatin 20mg tablets |
|  | 93620998 | Simvastatin 10mg tablets |
|  | 93871990 | Simvastatin 40mg tablets |
|  | 92409998 | Rosuvastatin 10mg tablets |
|  | 93243996 | Pravastatin 40mg tablets |
|  | 91194998 | Fluvastatin 80mg modified-release tablets |
|  | 93010990 | Colestyramine 4g oral powder sachets sugar free |
|  | 92549990 | Fenofibrate micronised 200mg capsules |
|  | 93244998 | Pravastatin 10mg tablets |
|  | 93244997 | Pravastatin 20mg tablets |
|  | 93244996 | Pravastatin 40mg tablets |
|  | 92539998 | Rosuvastatin 40mg tablets |
|  | 90310998 | Atorvastatin 80mg tablets |
|  | 93243997 | Pravastatin 20mg tablets |
|  | 92448998 | Cerivastatin 400microgram tablets |
|  | 94407990 | Simvastatin 20mg tablets |
|  | 93838990 | Bezafibrate 200mg tablets |
|  | 92471998 | Simvastatin 80mg tablets |
|  | 93851992 | Colestipol 5g granules sachets sugar free |
|  | 90309998 | Atorvastatin 80mg tablets |
|  | 92460998 | Fenofibrate micronised 160mg tablets |
|  | 91316998 | Colestyramine sugar free powder |
|  | 93243998 | Pravastatin 10mg tablets |
|  | 92292998 | Ezetimibe 10mg tablets |
|  | 92154990 | Simvastatin 20mg/5ml oral suspension sugar free |
|  | 93541998 | Colestyramine 4g oral powder sachets |
|  | 93542998 | Colestyramine 4g oral powder sachets sugar free |
|  | 92804997 | Fluvastatin 40mg capsules |
|  | 94189997 | Fenofibrate micronised 200mg capsules |
|  | 92293998 | Ezetimibe 10mg tablets |
|  | 90649998 | Fenofibrate 200mg capsules |
|  | 92805998 | Fluvastatin 20mg capsules |
|  | 92804998 | Fluvastatin 20mg capsules |
|  | 92805997 | Fluvastatin 40mg capsules |
|  | 93619996 | Simvastatin 40mg tablets |
|  | 92220998 | Simvastatin 80mg tablets |
|  | 90653998 | Colestyramine 4g oral powder sachets sugar free |
|  | 94188997 | Fenofibrate micronised 200mg capsules |
|  | 94112992 | Cholestyramine 325 mg cap |
|  | 94188998 | Fenofibrate 100mg capsule |
|  | 94189998 | Fenofibrate 100mg capsules |
|  | 92804996 | Fluvastatin 80mg modified-release tablets |
|  | 93619997 | Simvastatin 20mg tablets |
|  | 95480990 | Simvastatin 10mg tablets |
|  | 95479990 | Simvastatin 20mg tablets |
|  | 95952997 | Bezafibrate 400mg modified-release tablets |
|  | 95550990 | Simvastatin 20mg tablets |
|  | 95551990 | Simvastatin 10mg tablets |
|  | 94925998 | Eicosapentaenoic acid 170mg / Docosahexaenoic acid 115mg capsules |
|  | 95478990 | Simvastatin 40mg tablets |
|  | 95471990 | Simvastatin 40mg tablets |
|  | 95475990 | Simvastatin 20mg tablets |
|  | 94799998 | Fenofibrate micronised 160mg tablets |
|  | 96295997 | Gemfibrozil 600mg tablets |
|  | 95474990 | Simvastatin 40mg tablets |
|  | 95472990 | Simvastatin 20mg tablets |
|  | 95451990 | Simvastatin 10mg tablets |
|  | 95549990 | Simvastatin 40mg tablets |
|  | 96295998 | Gemfibrozil 300mg capsules |
|  | 94927990 | Simvastatin 80mg tablets |
|  | 94827992 | Colestyramine 4g oral powder sachets |
|  | 95501990 | Simvastatin 40mg tablets |
|  | 94782990 | Pravastatin 20mg tablets |
|  | 95185990 | Simvastatin 80mg tablets |
|  | 95494990 | Simvastatin 20mg tablets |
|  | 94605998 | Colestipol 5g granules sachets sugar free |
|  | 95495990 | Simvastatin 10mg tablets |
|  | 94851990 | Pravastatin 10mg tablets |
|  | 95500990 | Simvastatin 80mg tablets |
|  | 94830990 | Pravastatin 20mg tablets |
|  | 95502990 | Simvastatin 20mg tablets |
|  | 97078998 | Fish oil concentrate 1g capsules |
|  | 96685990 | Bezafibrate 400mg modified-release tablets |
|  | 96685989 | Bezafibrate 200mg tablets |
|  | 97377979 | Cerivastatin sodium 300mcg tablets |
|  | 94831990 | Pravastatin 10mg tablets |
|  | 97078997 | Fish oil concentrate oral liquid |
|  | 94661998 | Colestipol 5g granules sachets sugar free |
|  | 95482990 | Simvastatin 20mg tablets |
|  | 95483990 | Simvastatin 10mg tablets |
|  | 95486990 | Simvastatin 40mg tablets |
|  | 95508990 | Simvastatin 10mg tablets |
|  | 95487990 | Simvastatin 20mg tablets |
|  | 95952998 | Bezafibrate 200mg tablets |
|  | 97078996 | Fish oil concentrate oral emulsion |
|  | 94850990 | Pravastatin 20mg tablets |
|  | 94849990 | Pravastatin 40mg tablets |
|  | 94662998 | Colestipol 5g granules sachets sugar free |
|  | 95493990 | Simvastatin 40mg tablets |
|  | 95847990 | Colestyramine 4g oral powder sachets sugar free |
|  | 95098992 | Hexopal 200 mg tab |
|  | 97455979 | Pravastatin 10mg tablets |
|  | 95481990 | Simvastatin 40mg tablets |
|  | 94661997 | Colestipol 5g granules sachets sugar free |
|  | 97430979 | Fluvastatin 20mg capsules |
|  | 95805998 | Bezafibrate 400mg modified release tablets |
|  | 95405990 | Simvastatin 40mg tablets |
|  | 94789990 | Pravastatin 10mg tablets |
|  | 95401998 | Probucol 250mg tablet |
|  | 97247997 | Gemfibrozil 600mg tablets |
|  | 97247998 | Gemfibrozil 300mg capsules |
|  | 96134990 | Colestyramine 4g oral powder sachets |
|  | 95278990 | Simvastatin 20mg tablets |
|  | 95277990 | Simvastatin 40mg tablets |
|  | 95372990 | Simvastatin |

**Read codes for Excessive Alcohol Intake**

| **Alcohol Excess** | **READ codes** | **Description** |
| --- | --- | --- |
|  | 1365.00 | Heavy drinker - 7-9u/day |
|  | 1366.00 | Very heavy drinker - >9u/day |
|  | 136a.00 | Increasing risk drinking |
|  | 136b.00 | Feels should cut down drinking |
|  | 136c.00 | Higher risk drinking |
|  | 136D.00 | Ex-heavy drinker - (7-9u/day) |
|  | 136E.00 | Ex-very heavy drinker-(>9u/d) |
|  | 136K.00 | Alcohol intake above recommended sensible limits |
|  | 136O.00 | Moderate drinker |
|  | 136P.00 | Heavy drinker |
|  | 136Q.00 | Very heavy drinker |
|  | 136R.00 | Binge drinker |
|  | 136S.00 | Hazardous alcohol use |
|  | 136T.00 | Harmful alcohol use |
|  | 136W.00 | Alcohol misuse |
|  | 136Y.00 | Drinks in morning to get rid of hangover |
|  | 1462.00 | H/O: alcoholism |
|  | 1B1c.00 | Alcohol induced hallucinations |
|  | 2126C00 | Alcohol dependence resolved |
|  | 38P0300 | HoNOSCA item 4 - alcohol, substance/solvent misuse |
|  | 7P22100 | Delivery of rehabilitation for alcohol addiction |
|  | 8BA8.00 | Alcohol detoxification |
|  | 8CAM000 | Advised to abstain from alcohol consumption |
|  | 8CAv.00 | Advised to contact primary care alcohol worker |
|  | 8CdK.00 | Specialist alcohol treatment service signposted |
|  | 8G32.00 | Aversion therapy - alcoholism |
|  | 8H35.00 | Admitted to alcohol detoxification centre |
|  | 8H7p.00 | Referral to community alcohol team |
|  | 8HHe.00 | Referral to community drug and alcohol team |
|  | 8HkG.00 | Referral to specialist alcohol treatment service |
|  | 8HkJ.00 | Referral to alcohol brief intervention service |
|  | 8IAF.00 | Brief intervention for excessive alcohol consumptn declined |
|  | 8IAJ.00 | Declined referral to specialist alcohol treatment service |
|  | 8IAt.00 | Extended interven for excessive alcohol consumption declined |
|  | 8IEA.00 | Referral to community alcohol team declined |
|  | 8W2..00 | Refer to MH services deferred until alcohol misuse resolved |
|  | 9k1..00 | Alcohol misuse - enhanced services administration |
|  | 9k11.00 | Alcohol consumption counselling |
|  | 9k12.00 | Alcohol misuse - enhanced service completed |
|  | 9k14.00 | Alcohol counselling by other agencies |
|  | 9k1A.00 | Brief intervention for excessive alcohol consumptn completed |
|  | 9k1B.00 | Extended intervention for excessive alcohol consumptn complt |
|  | 9NJz.00 | In-house alcohol detoxification |
|  | 9NN2.00 | Under care of community alcohol team |
|  | 9NzA.00 | Hospital attendance related to personal alcohol consumption |
|  | 9NzA.00 | Hospital attendance related to personal alcohol consumption |
|  | C150500 | Alcohol-induced pseudo-Cushing's syndrome |
|  | E01..00 | Alcoholic psychoses |
|  | E010.00 | Alcohol withdrawal delirium |
|  | E010.11 | DTs - delirium tremens |
|  | E010.12 | Delirium tremens |
|  | E011.00 | Alcohol amnestic syndrome |
|  | E011000 | Korsakov's alcoholic psychosis |
|  | E011100 | Korsakov's alcoholic psychosis with peripheral neuritis |
|  | E011200 | Wernicke-Korsakov syndrome |
|  | E011z00 | Alcohol amnestic syndrome NOS |
|  | E012.00 | Other alcoholic dementia |
|  | E012.11 | Alcoholic dementia NOS |
|  | E012000 | Chronic alcoholic brain syndrome |
|  | E013.00 | Alcohol withdrawal hallucinosis |
|  | E014.00 | Pathological alcohol intoxication |
|  | E014.11 | Drunkenness - pathological |
|  | E015.00 | Alcoholic paranoia |
|  | E01y.00 | Other alcoholic psychosis |
|  | E01y000 | Alcohol withdrawal syndrome |
|  | E01yz00 | Other alcoholic psychosis NOS |
|  | E01z.00 | Alcoholic psychosis NOS |
|  | E23..00 | Alcohol dependence syndrome |
|  | E23..11 | Alcoholism |
|  | E23..12 | Alcohol problem drinking |
|  | E230.00 | Acute alcoholic intoxication in alcoholism |
|  | E230.11 | Alcohol dependence with acute alcoholic intoxication |
|  | E230000 | Acute alcoholic intoxication, unspecified, in alcoholism |
|  | E230100 | Continuous acute alcoholic intoxication in alcoholism |
|  | E230200 | Episodic acute alcoholic intoxication in alcoholism |
|  | E230300 | Acute alcoholic intoxication in remission, in alcoholism |
|  | E230z00 | Acute alcoholic intoxication in alcoholism NOS |
|  | E231.00 | Chronic alcoholism |
|  | E231.11 | Dipsomania |
|  | E231000 | Unspecified chronic alcoholism |
|  | E231100 | Continuous chronic alcoholism |
|  | E231200 | Episodic chronic alcoholism |
|  | E231300 | Chronic alcoholism in remission |
|  | E231z00 | Chronic alcoholism NOS |
|  | E23z.00 | Alcohol dependence syndrome NOS |
|  | E250.00 | Nondependent alcohol abuse |
|  | E250.11 | Drunkenness NOS |
|  | E250.12 | Hangover (alcohol) |
|  | E250.13 | Inebriety NOS |
|  | E250.14 | Intoxication - alcohol |
|  | E250000 | Nondependent alcohol abuse, unspecified |
|  | E250100 | Nondependent alcohol abuse, continuous |
|  | E250200 | Nondependent alcohol abuse, episodic |
|  | E250300 | Nondependent alcohol abuse in remission |
|  | E250z00 | Nondependent alcohol abuse NOS |
|  | Eu10.00 | [X]Mental and behavioural disorders due to use of alcohol |
|  | Eu10000 | [X]Mental & behav dis due to use alcohol: acute intoxication |
|  | Eu10011 | [X]Acute alcoholic drunkenness |
|  | Eu10100 | [X]Mental and behav dis due to use of alcohol: harmful use |
|  | Eu10200 | [X]Mental and behav dis due to use alcohol: dependence syndr |
|  | Eu10211 | [X]Alcohol addiction |
|  | Eu10212 | [X]Chronic alcoholism |
|  | Eu10213 | [X]Dipsomania |
|  | Eu10300 | [X]Mental and behav dis due to use alcohol: withdrawal state |
|  | Eu10400 | [X]Men & behav dis due alcohl: withdrawl state with delirium |
|  | Eu10411 | [X]Delirium tremens, alcohol induced |
|  | Eu10500 | [X]Mental & behav dis due to use alcohol: psychotic disorder |
|  | Eu10511 | [X]Alcoholic hallucinosis |
|  | Eu10512 | [X]Alcoholic jealousy |
|  | Eu10513 | [X]Alcoholic paranoia |
|  | Eu10514 | [X]Alcoholic psychosis NOS |
|  | Eu10600 | [X]Mental and behav dis due to use alcohol: amnesic syndrome |
|  | Eu10611 | [X]Korsakov's psychosis, alcohol induced |
|  | Eu10700 | [X]Men & behav dis due alcoh: resid & late-onset psychot dis |
|  | Eu10711 | [X]Alcoholic dementia NOS |
|  | Eu10712 | [X]Chronic alcoholic brain syndrome |
|  | Eu10800 | [X]Alcohol withdrawal-induced seizure |
|  | Eu10y00 | [X]Men & behav dis due to use alcohol: oth men & behav dis |
|  | Eu10z00 | [X]Ment & behav dis due use alcohol: unsp ment & behav dis |
|  | F11x000 | Cerebral degeneration due to alcoholism |
|  | F11x011 | Alcoholic encephalopathy |
|  | F144000 | Cerebellar ataxia due to alcoholism |
|  | F25B.00 | Alcohol-induced epilepsy |
|  | F375.00 | Alcoholic polyneuropathy |
|  | F394100 | Alcoholic myopathy |
|  | G555.00 | Alcoholic cardiomyopathy |
|  | G852300 | Oesophageal varices in alcoholic cirrhosis of the liver |
|  | J153.00 | Alcoholic gastritis |
|  | J610.00 | Alcoholic fatty liver |
|  | J611.00 | Acute alcoholic hepatitis |
|  | J612.00 | Alcoholic cirrhosis of liver |
|  | J612000 | Alcoholic fibrosis and sclerosis of liver |
|  | J613.00 | Alcoholic liver damage unspecified |
|  | J613000 | Alcoholic hepatic failure |
|  | J617.00 | Alcoholic hepatitis |
|  | J617000 | Chronic alcoholic hepatitis |
|  | J670800 | Alcohol-induced acute pancreatitis |
|  | J671000 | Alcohol-induced chronic pancreatitis |
|  | R103.00 | [D]Alcohol blood level excessive |
|  | SM0..00 | Alcohol causing toxic effect |
|  | SM00z00 | Ethyl alcohol causing toxic effect NOS |
|  | SM0z.00 | Alcohol causing toxic effect NOS |
|  | Z191.00 | Alcohol detoxification |
|  | Z191100 | Alcohol withdrawal regime |
|  | Z191111 | Drying out |
|  | Z191200 | Planned reduction of alcohol consumption |
|  | Z191211 | Alcohol reduction programme |
|  | Z191300 | Controlled drinking regime |
|  | Z191400 | Self-monitoring of alcohol intake |
|  | ZC22200 | Advice to change alcoholic drink intake |
|  | 66e..00 | Alcohol disorder monitoring |
|  | 66e0.00 | Alcohol abuse monitoring |

**Read codes for hypothyroidism**

| **Hypothyroidism** | **READ**  **Codes** | **Description** |
| --- | --- | --- |
|  | C03..00 | Congenital hypothyroidism |
|  | C03..11 | Cretinism |
|  | C030.00 | Pendred's syndrome |
|  | C031.00 | Goitrous cretin |
|  | C03y.00 | Other specified congenital hypothyroidism |
|  | C03y000 | Congenital hypothyroidism with diffuse goitre |
|  | C03y100 | Congenital hypothyroidism without goitre |
|  | C03z.00 | Congenital hypothyroidism NOS |
|  | C03z.11 | Congenital thyroid insufficiency |
|  | C03z.12 | Cretinism |
|  | C04..00 | Acquired hypothyroidism |
|  | C04..11 | Myxoedema |
|  | C04..12 | Thyroid deficiency |
|  | C04..13 | Hypothyroidism |
|  | C040.00 | Postsurgical hypothyroidism |
|  | C040.11 | Post ablative hypothyroidism |
|  | C041.00 | Other postablative hypothyroidism |
|  | C041000 | Irradiation hypothyroidism |
|  | C041z00 | Postablative hypothyroidism NOS |
|  | C042.00 | Iodine hypothyroidism |
|  | C043.00 | Other iatrogenic hypothyroidism |
|  | C043000 | Hypothyroidism resulting from para-aminosalicylic acid |
|  | C043100 | Hypothyroidism resulting from phenylbutazone |
|  | C043200 | Hypothyroidism resulting from resorcinol |
|  | C043z00 | Iatrogenic hypothyroidism NOS |
|  | C044.00 | Postinfectious hypothyroidism |
|  | C045.00 | Acquired atrophy of thyroid |
|  | C046.00 | Autoimmune myxoedema |
|  | C047.00 | Subclinical hypothyroidism |
|  | C04y.00 | Other acquired hypothyroidism |
|  | C04z.00 | Hypothyroidism NOS |
|  | C04z.11 | Pretibial myxoedema - hypothyroid |
|  | C04z.12 | Thyroid insufficiency |
|  | C04z.13 | Hypothyroid goitre, acquired |
|  | C04z000 | Premature puberty due to hypothyroidism |

**Read codes for NAFLD**

| **Non-alcoholic fatty liver disease** | **READ**  **Codes** | **Description** |
| --- | --- | --- |
|  | J61y800 | Non-alcoholic steatohepatitis |
|  | J61y100 | Non-alcoholic fatty liver |

**APPENDIX 2. Sensitivity Analyses Table 1.** Association between body size phenotype and metabolic status and NAFLD including those with missing data for smoking status and Townsend score

| **Body size phenotype and metabolic status** |  |  |
| --- | --- | --- |
|  | **Unadjusted HR** | **Adjusted HR** |
| Underweight, 0 metabolic abnormalities | 0.51 (0.35-0.74) | 0.53 (0.36-0.77) |
| Underweight, ≥ 1 metabolic abnormality | 1.28 (0.69-2.39) | 0.95 (0.50-1.78) |
| Normal weight, 0 metabolic abnormalities | 1.00 (Ref) | 1.00 (Ref) |
| Normal weight, 1 metabolic abnormality | 2.39 (2.14-2.68) | 2.25 (1.97-2.57) |
| Normal weight, ≥ 2 metabolic abnormalities | 2.82 (2.45-3.24) | 2.35 (1.98- 2.79) |
| Overweight, 0 metabolic abnormalities | 3.35 (3.11-3.61) | 3.18 (2.95-3.43) |
| Overweight, 1 metabolic abnormality | 6.03 (5.57-6.53) | 7.17 (6.49-7.91) |
| Overweight, ≥ 2 metabolic abnormalities | 6.95 (6.40-7.56) | 9.44 (8.36-10.66) |
| Obese, 0 metabolic abnormalities | 7.11 (6.61-7.65) | 6.78 (6.30-7.29) |
| Obese, 1 metabolic abnormality | 10.73 (9.95-11.57) | 11.91 (10.93-12.99) |
| Obese, ≥ 2 metabolic abnormalities | 12.95 (12.03-13.95) | 17.25 (15.67-19.01) |

**Table 2.**  Development of NAFLD by body size phenotype and metabolic status using metabolic status derived from diagnostic codes for diabetes and hypertension and prescription records of lipid-modifying drugs only

| **Body size phenotype and metabolic status** |  |  |  |  |  |  |
| --- | --- | --- | --- | --- | --- | --- |
|  | **Sample size** | **Incident Cases** | **Person-years** | **Incidence rate (per 1,000 person-years)** | **Unadjusted HR** | **Adjusted HR** |
| Underweight, 0 metabolic abnormalities | 97328 | 25 | 432766.9 | 0.06 | 0.44 (0.29-0.65) | 0.46 (0.31-0.69) |
| Underweight, ≥ 1 metabolic abnormality | 12756 | 5 | 51713.7 | 0.1 | 0.79 (0.33-1.90) | 0.55 (0.23-1.34) |
| Normal weight, 0 metabolic abnormalities | 1451488 | 1074 | 7596935 | 0.14 | 1.00 (Ref) | 1.00 (Ref) |
| Normal weight, 1 metabolic abnormality | 170872 | 254 | 1028727 | 0.25 | 1.72 (1.50-1.97) | 1.45 (1.24-1.70) |
| Normal weight, ≥ 2 metabolic abnormalities | 84572 | 150 | 457973.5 | 0.33 | 2.46 (2.07-2.92) | 1.75 (1.44- 2.13) |
| Overweight, 0 metabolic abnormalities | 983606 | 2826 | 5671643 | 0.5 | 3.47 (3.23-3.72) | 3.25 (3.03-3.49) |
| Overweight, 1 metabolic abnormality | 237003 | 1116 | 1553047 | 0.72 | 4.94 (4.54-5.37) | 5.47 (4.90-6.10) |
| Overweight, ≥ 2 metabolic abnormalities | 157287 | 765 | 937880.7 | 0.82 | 5.89 (5.37-6.47) | 5.68 (4.99-6.47) |
| Obese, 0 metabolic abnormalities | 575933 | 3451 | 3327632 | 1.04 | 7.27 (6.79-7.79) | 6.88 (6.42-7.37) |
| Obese, 1 metabolic abnormality | 190534 | 1691 | 1257030 | 1.35 | 9.23 (8.55-9.97) | 10.14 (9.24-11.12) |
| Obese, ≥ 2 metabolic abnormalities | 159670 | 1510 | 938834.2 | 1.61 | 11.62 (10.74-12.57) | 12.68 (11.41-14.1) |
